# Eukaryotic gene regulation at equilibrium, or non?

**DOI:** 10.1016/j.coisb.2022.100435

**Published:** 2022-10-20

**Authors:** Benjamin Zoller, Thomas Gregor, Gašper Tkačik

**Affiliations:** 1 Lewis-Sigler Institute for Integrative Genomics, Princeton University, Princeton, NJ, USA; 2 Joseph Henry Laboratories of Physics, Princeton University, Princeton, NJ, USA; 3 Department of Developmental and Stem Cell Biology UMR3738, Institut Pasteur, Paris, France; 4 Institute of Science and Technology Austria, Klosterneuburg, Austria

**Keywords:** Gene regulation, Optimization, Non-equilibrium regulation, Modeling

## Abstract

Models of transcriptional regulation that assume equilibrium binding of transcription factors have been less successful at predicting gene expression from sequence in eukaryotes than in bacteria. This could be due to the non-equilibrium nature of eukaryotic regulation. Unfortunately, the space of possible non-equilibrium mechanisms is vast and predominantly uninteresting. The key question is therefore how this space can be navigated efficiently, to focus on mechanisms and models that are biologically relevant. In this review, we advocate for the normative role of theory—theory that prescribes rather than just describes—in providing such a focus. Theory should expand its remit beyond inferring mechanistic models from data, towards identifying non-equilibrium gene regulatory schemes that may have been evolutionarily selected, despite their energy consumption, because they are precise, reliable, fast, or otherwise outperform regulation at equilibrium. We illustrate our reasoning by toy examples for which we provide simulation code.

## Non-equilibrium processes essential to life consume energy

A defining physical property of life is its non-equilibrium nature (a cell at thermodynamic equilibrium with its surrounding is typically a dead cell). Indeed, living organisms are characterized as thermodynamically open systems involving the exchange of heat, work and matter with the environment, in order to achieve self-organization, growth, homeostasis, adaptation, and so on. The non-equilibrium activity of cells is typically driven by internal enzymatic processes that hydrolize ATP or other donors of covalent modification rather than by external forces [1].

In addition to the exchange of matter, cells also carefully orchestrate flows of information, by tying their internal chemical state to changes in the environment [2]. Typical examples include the chemotaxis network of *E. coli*, where information about chemoattractant gradient is detected by receptors at the cell surface and transduced to the motors driving the flagella [3], and MAPK signaling pathways in yeast, where information propagates through phosphorylation in a cascade of signaling molecules before affecting gene expression [4]. Chemical modifications at the heart of these pathways consume ATP and are thus intrinsically kept far from equilibrium. More generally, information processing in biological systems can be energetically costly, especially when speed and precision are at a premium, as substantiated by well-known examples in neuroscience [5,6].

In contrast, gene regulation has historically been studied through the lens of chemical processes at equilibrium, which are greatly successful in prokaryotes. There, the “thermodynamic model of gene regulation” assumes that the equilibrium occupancy of transcription factors (TFs) on their regulatory sites on the DNA dictates downstream gene expression, and this provides a dramatic conceptual and predictive simplification of the regulatory processes [7]. With some notable exceptions [8], thermodynamic models in bacteria have enabled a concordance between *in vivo* and *in vitro* as well as between steady-state and kinetic measurements [9–12].

But why should nature limit itself to equilibrium regulatory processes? We stress that what we denominate here as equilibrium (EQ) versus non-equilibrium (NEQ) depends on the boundaries of the system under consideration. It is clear that transcription as a whole is a NEQ process, as energy is expended by the enzyme RNA polymerase (RNAP) to synthetize the RNA polymer. What we are focusing on here are the regulatory steps preceding RNAP activity. These steps involve the TFs that govern the likelihood of RNAP binding and unbind and thus *control* productive transcription. With this clarification in mind, the key argument for regulation at equilibrium is either one of modeling simplicity (Ockham’s razor, time-scale separation, coarse-graining) [10] or one of energy expenditure minimization [3] – which could be evolutionary selected for.

Given our current estimates, however, the energetic costs of transcriptional regulation should be negligible compared to other known energy-consuming cellular processes. The average cell in the human body can produce between 10^8^–10^9^ ATP/s, of which motility consumes 10^5^–10^6^ ATP/s and protein production more than 10^7^ ATP/s (by maintaining typical protein concentrations) [13]. The overall metabolic cost to maintain a eukaryotic cell is around 10^7^ ATP/s, while making a new daughter cell costs around 10^12^ ATP in total. Overall, the cost of DNA replication is one to two orders of magnitude smaller than the cost of transcription (synthesis and degradation), which is a further one to two orders of magnitude smaller than the cost of translation (synthesis and degradation) [14]. The amount of ATP needed to support the actin cytoskeleton has been estimated to be around 50% of the total ATP consumption of a cell [15]. Thus, the vast majority of energy consumed by a cell appears dedicated to protein renewal and cytoskeleton rearrangement. Taken together, it is difficult to imagine how any putative ATP costs of regulation and initiation (which are most likely smaller than the follow-up cost of productive transcription and translation) could constitute a relevant dent in the cell’s total energy budget. We should therefore turn our attention to other costs and benefits of non-equilibrium regulatory schemes.

In the following sections we briefly review the basic NEQ mechanisms and their possible regulatory benefits. Then we give an overview of current evidence for NEQ regulation in eukaryotes, and highlight the necessity care required to properly assess which signatures support or rule out NEQ and EQ mechanisms. By means of toy models we demonstrate that the space of NEQ regulatory models is vast, but only a fraction of this space leads to functionally relevant regulatory phenotypes. Lastly, we propose one particular option to navigate the complexity inherent to NEQ models, by postulating that regulatory mechanisms evolved to optimize some of these regulatory phenotypes. This normative approach can generate both functional (what has been selected) and mechanistic (how it works) hypotheses, thus bringing together aspects of evolutionary and molecular biology.

## Fundamentals of non-equilibrium regulatory processes

To highlight the possible regulatory benefits of NEQ mechanisms, we adopt a biophysically-rooted kinetic description of regulatory processes [16]. Within that framework, equilibrium has a very specific meaning, namely the thermodynamic equilibrium of the underlying molecular processes, which can be traced back to their time-reversibility. These qualifications are important. First, it is possible to consider truly NEQ (kinetic) processes but coarse-grain them such that their effective description resembles EQ [10,17,18]; this is not our focus here. Second, it is important to differentiate between free energy dissipation during a transient relaxation to the steady state which can be discussed also for EQ systems, and dissipation in the true NEQ steady state which is unique to NEQ systems; here, we will mainly discuss the latter. In what follows, we employ a general “linear framework” based on the linear chemical master equation. It describes the relevant processes in terms of chemical reaction networks [19–22] and defines their degree of time-irreversibility (i.e. their non-equilibrium character) through the violation of detailed balance.

Most reaction networks are NEQ. Indeed, barring very particular network topologies (such as sequences or trees), a random assignment of reaction rates between the vertices of the network will inevitably yield a NEQ system. This is because EQ models must implement strict relationships between their reactions rates to satisfy detailed balance, encoding for the reversibility of elementary processes [1]. By breaking detailed balance, one can access the full reaction network space, and, in particular, irreversible reaction cycles, the simplest NEQ structures which imply energy expenditure and dissipation [19–21].

**Figure 1:**
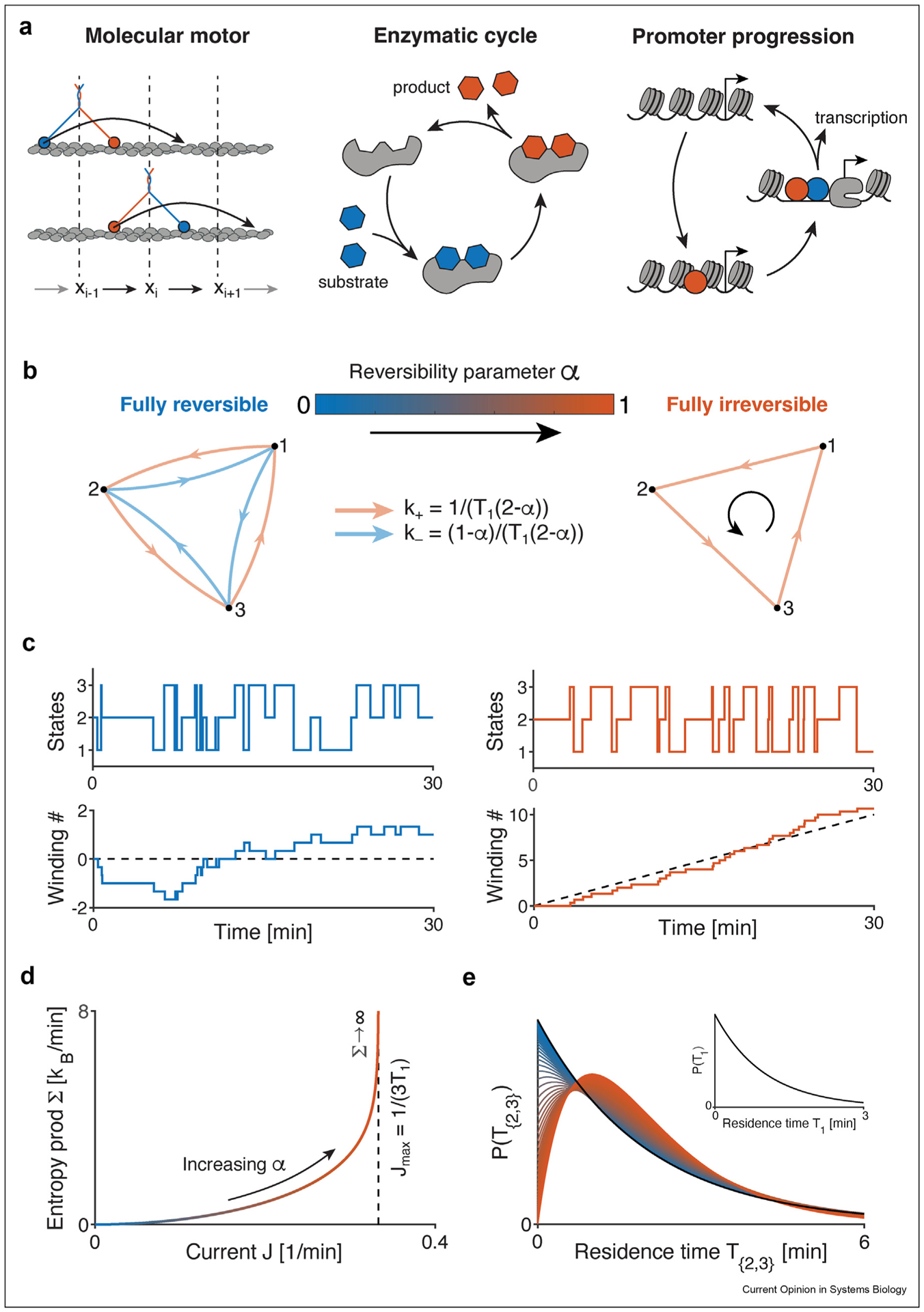
Reaction cycles as the fundamental “unit” of NEQ models highlight key signatures of time-irreversibility. **(a)** Three biological examples of irreversible cycles. (Left) A walking myosin on an actin filament. (Middle) An enzymatic cycle. (Right) Gene promoter progression towards activation. **(b)** A class of models that can continuously transform from EQ to NEQ is parametrized by a unique “reversibility” parameter α for a given size (N = 3 states), at constant mean state occupancies (P_i_ = 1/N) and residence times (T_i_ = T_1_ = 1min). **(c)** Two stochastic realizations, for a fully reversible (α = 0, left) and fully irreversible cycle (α = 1, right). (Top) Individual state occupancies. (Bottom) Winding number for each realization, i.e., the number of counter-clockwise cycle completions. Irreversibility leads to a clear temporal ordering of reactions, as highlighted by the progression of the winding number. **(d)** Entropy production Σ or dissipation (Σ times temperature) as a function of the current J along the cycle. The current J is the slope of the mean winding number (black dash line in C), whose magnitude is equal to the inverse of the period of the cycle. Both J and Σ monotonically increase as the cycle approaches full irreversibility (α = 1). When α = 1, the current is maximal, J_max_ = 1/(NT_1_) and the entropy production tends to infinity. The presence of currents and entropy production are hallmarks of NEQ reaction schemes. **(e)** Residence time distribution P(T_{2,3}_) for the combined states 2 and 3, i.e., time spent in 2 and/or 3 before ending in 1. T_{2,3}_ is phase-type distributed and its shape depends on α, changing from exponential-like (α = 0) to “peaked” (α = 1). Peaked residence time distributions are another signature of strongly irreversible processes. (Inset) residence time distribution P(T_1_) for individual states. T_1_ is exponentially distributed and does not depend on α.

Cycles have been widely used to describe molecular motors, phosphorylation cycles (cell cycle, circadian cycle) [23], transition state cycle of enzymes, and regulation by gene promoters (promoter progression) [24,25] (Figure 1a–b). In particular, for transcriptional regulation, cycles have been shown to be sufficiently rich to implement a vast range of logical computations [26]. NEQ reaction networks also permit regulatory mechanisms with improved or novel functionality (see below). They have been the focus of several recent publications: improved fidelity and specificity (via proof-reading mechanisms) [27–30], gene expression noise reduction [31], improved information transmission [32], improved sensitivity (ultra-sensitivity, high Hill coefficients of the regulatory functions) [33,34], improved sensing (beyond Berg-Purcell limit) [35], faster relaxation [36,37], and improved timing precision [38,39].

## Direct experimental signatures of non-equilibrium regulation

The two most unequivocal signatures of NEQ processes at steady state are heat dissipation with entropy production and the irreversibility of the underlying reactions (Figure 1b & d). These two features are intimately linked, since at least in principle knowing the amount of dissipated heat is informative about the degree of reversibility and vice versa [20,40]. Estimating energy expenditure in absolute units from chemical reaction models is a priori possible, but very challenging to apply in practice. As a first complication, one must know the exact degrees of freedom (the microstates) of the system to connect dissipation with kinetics. Missing microscopic details will bias the estimation since coarse-graining of the reaction network reduces the apparent dissipation [18]. Moreover, defining an adequate temperature for a molecular non-equilibrium system can be non-trivial.

Recent theoretical progress circumvents some of these issues, e.g., by quantifying fluxes in configurational phase space [41], by quantifying the irreversibility of fluctuations in timing of events from time series using semi-Markov processes [42], and by bounding the entropy production from time series using the waiting time statistics of hidden-Markov process [43].

Overall, if a system is observed to violate detailed balance at any scale, then the underlying molecular mechanism has to be out of equilibrium, enabling us to identify a NEQ process [44]. Despite these theoretical advances, measuring heat dissipation in practice remains challenging and has been typically limited to whole organisms in the context of metabolism [45,46], or to *in vitro* molecular systems of limited complexity, such as molecular motors and enzymatic reactions [47]. Such measurements for complex *in vivo* processes such as gene regulation are currently out of reach.

Irreversibility, however, can have consequences other than heat dissipation, which can be inferred from time series measurements of observables even in complex *in vivo* systems (Figure 1c). For example, provided that the states of the system can be determined from data (such as configurations of the molecular motor, gene activity, etc.), one can estimate the distribution of residence times (times spent in a set of states, also known as dwell or waiting times), whose shape depends on the reversibility of the underlying reactions (Figure 1e). It has been demonstrated that peaked, gamma-like distributions are a strong signature of irreversibility, and thus of NEQ processes [33,48].

Notably, the silent intervals between multiple rounds of transcriptional bursts in eukaryotic genes are well described by such peaked distributions [49,50], suggesting that the inactive periods of these genes arise from multiple irreversible sequential steps required before transcriptional initiation. Such a succession of irreversible steps leads to a refractory period [24,51], a minimal duration preventing immediate reactivation of transcription. The nature of these refractory periods and the underlying sequential steps remain poorly understood, and likely depend on the induction pathway. The steps could be related to sequential modifications of the chromatin template mediated by various transcription and pioneer factors, the establishment of contact between regulatory elements and promoter through chromatin looping, and the resulting formation of cofactor condensates leading to the assembly of the pre-initiation complex. New evidence suggests that transcription initiation stimulates cofactor condensates, whereas bursts stimulate dissolution, implementing a NEQ feedback [52]. Such a negative feedback should typically lead to pulsatile bursts of transcription whose silent interval distributions are again peaked rather than exponential [53].

## Regulatory phenotypes bearing indirect signatures of non-equilibrium regulation

**Figure 2:**
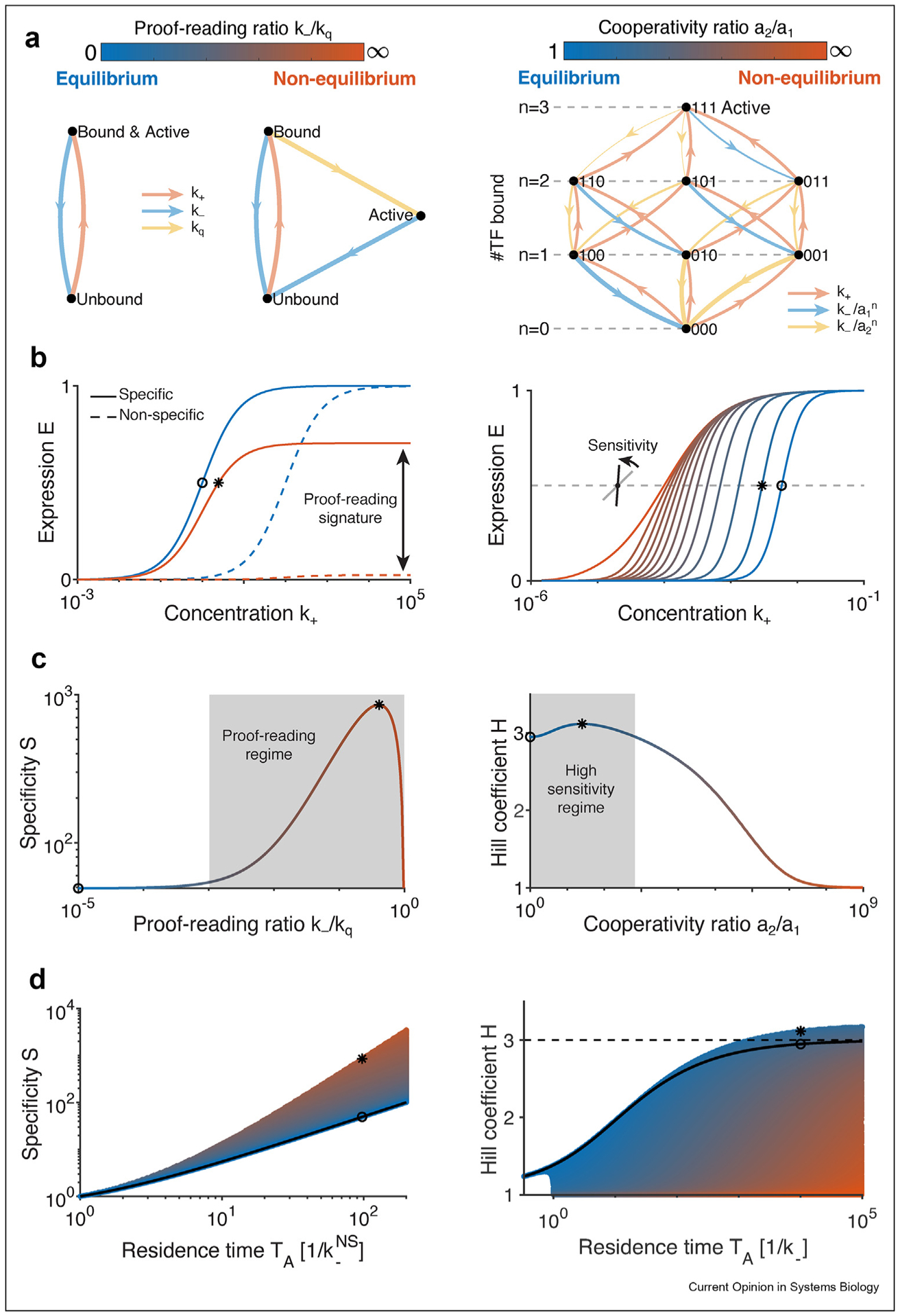
Two toy models of gene regulation with functional advantages when operating out-of-equilibrium (NEQ) versus in equilibrium (EQ). **(a)** (Left) High-specificity model implements kinetic proof-reading through an irreversible transition with rate k_q_ leading to the active state (expression E is equal to active state occupancy). The two other states denote TF bound/unbound to the DNA. Ratio k_−_/k_q_ determines the strength of the proof-reading. When k_q_→∞, the model reduces to the simple two state model (unbound and active) at equilibrium. (Right) High-sensitivity model with N = 3 TF binding sites (8 occupancy states) and higher-order cooperativity ${\text{a}}_{\text{i}}^{\text{n}}$ between TFs (with a_2_ ≥ a_1_ ≥ 1). The a_i_ are interpreted as cooperativities as they reduce the TF unbinding rates in presence of n other bound TFs. Expression E is “all-or-nothing”, equal to the occupancy of the all-bound state (active state). Detailed balance holds only when a_1_ = a_2_; the ratio a_2_/a_1_ moves the model out-of-equilibrium by controlling the degree of asymmetry among cooperativities. For both models, the TF binding rate k_+_ is assumed to be proportional to the TF concentration. **(b)** Induction curves. (Left) Induction curves for EQ (blue) and NEQ (orange) proof-reading model with specific TF binding site (k_−_ = 1) and unspecific site (k_−_^NS^ = 10^2^). Both models lead to similar induction curves for specific sites, but NEQ model’s expression plateaus at k_q_/(k_−_ + k_q_) for non-specific binding whereas the EQ model still plateaus at 1. (Right) Induction for EQ (blue) and NEQ (blue to orange) asymmetric cooperativity models (with same residence time T_A_). The NEQ models achieve a vast range of sensitivities, defined as the slope at half-maximum expression. **(c)** Optimal operating regime of the two models. (Left) Specificity S, defined as the ratio of expression from specific and non-specific TF binding sites, as a function of the proof-reading ratio k_−_/k_q_, with TF concentration adjusted to hold E = 0.5, shows an optimal regime (10^−3^ < k_−_/k_q_ < ≃ 1, gray region) where the specificity of a NEQ model clearly outperforms the EQ limit, k_q_→∞. (Right) Hill coefficient H, defined as the log-derivative of the expression curve at half maximum, as a function of the cooperativity ratio a_2_/a_1_, with E = 0.5, and fixed active state residence time, shows an optimal regime (1 < a_2_/a_1_ < f(a_1_), gray region) where the sensitivity of a NEQ model is larger than the EQ limit, a_1_ = a_2_. **(d)** Only a fraction of regulatory phenotype space is accessible at fixed average expression. (Left) All NEQ models outperform the EQ limit (black curve), though the maximal specificity increase is limited. (Right) A fraction of NEQ models outperforms the EQ limit (black curve), with some breaking the limit of H = N = 3 set by Hill-type regulation.

Other, even more circumstantial signatures of NEQ regulation can be derived from response properties of enhancers or promoters, i.e., from their “regulatory phenotypes”. For example, one can focus on gene regulatory functions (also known as input-output relations or induction curves, showing the gene expression level as a function of inducer or regulator concentration), and in particular on their specificity or sensitivity (Figure 2) [34,54]. One can argue that NEQ schemes make it possible to achieve a certain high level of specificity or sensitivity, and in this context, recent measurements in eukaryotes tend to favor NEQ regulation schemes [55,56]. Such observations, however, do not constitute a definite proof. This is because even if current evidence is consistent with a proposed NEQ scheme, it typically does not rule out the existence of alternative EQ mechanisms which would be equally consistent with measured regulatory phenotypes.

We illustrate this discussion by building toy models that achieve two desired regulatory phenotypes, one for high specificity (Figure 2a, left) and another for high sensitivity (Figure 2a, right). The high-specificity model achieves good discrimination between specific and non-specific TF activation by using a NEQ kinetic proof-reading scheme [27,28], thereby outperforming alternatives at equilibrium (Figure 2b and c, left). The high-sensitivity model can achieve a steep induction curve (high apparent Hill coefficient) by using NEQ asymmetric higher-order cooperativities, outperforming alternatives at equilibrium for a small range of NEQ cooperativities (Figure 2b and c, right). These examples demonstrate that NEQ mechanisms can (but don’t always) provide functional advantages over equivalent EQ models that utilize the same number of TF binding sites and expression levels (Figure 2c, d).

Despite the attractiveness of these theoretical ideas, identifying NEQ mechanisms from experimentally measured induction curves can be challenging, as their signatures can be subtle or ambiguous (especially when focusing on individual regulatory phenotypes). Proof-reading could plausibly be detected from the difference in the induction curve plateaus for specific and non-specific TFs (or alternatively from cognate and non-cognate, i.e. mutated, TF binding sites) (Figure 2b). Detecting higher-order cooperativities necessitates measuring the whole induction curve precisely over a large range of input TF concentrations in a setup where the relevant TF binding sites are fully known. Neither of these tasks appears easy, especially if the predicted effects are not large or if there are substantial systematic uncertainties about the experimental setup.

Recent work has systematically dissected the transcriptional response of a eukaryotic enhancer in the fly embryo to demonstrate the limitations of EQ pairwise cooperativity that is thought to prevail in bacteria, and argue that NEQ higher-order cooperativities between transcription factors and cofactors (such as pioneer factors, Mediator, histone modifiers, etc.) are required to shape the transcriptional response [55]. Beyond steady state induction curves, one could look at the population-level distributions or temporally-resolved measurements. Distributions of mRNA or protein counts can be estimated using fixed approaches (smFISH, immuno-staining) [57]. Although full distributions are obviously more informative than their means, they may still be insufficient, however, to definitely distinguish EQ from NEQ mechanisms. Indeed, a steady state distribution can look almost identical (cf. Figure 1c, the steady state occupancies are identical across all models) or bear very subtle NEQ signatures [31], leading to issues of structural (infinite amount of data cannot discriminate models) or practical identifiability (large amount of data needed to discriminate models).

A better approach is to measure these population distributions along a time course after induction (during relaxation to steady state) [58]. Indeed, for dynamic data already at the level of the means typical signatures of NEQ processes are detectable, e.g. humps in the relaxation curves [37]. Recent work has demonstrated that thermodynamic models of TF binding have difficulties recapitulating the onset and relaxation dynamics for the expression of a eukaryotic gene, while a simple NEQ model of transcription-factor-driven sequential chromatin accessibility accounted for the data better [56]. Even here difficulties abound, mainly because temporal correlations among individual cells are lost in a population measurement, and – as explained in the second section – NEQ signatures are principally exposed through the sequential nature of state transitions in individual systems.

Analyzing temporal fluctuations from single-cell time-series is potentially the most powerful way to discriminate EQ vs NEQ models of regulation [24,25,49,50]. One possible approach would harness auto-correlation functions and waiting time distributions. While auto-correlation functions can be computed directly from gene expression time series using various imaging reporter schemes [59], waiting time distributions necessitate modeling of the time series, typically using Hidden Markov Models, to identify regulatory states of the promoter [25,49]. Recent advances in live imaging enable better identification of such states, by measuring both input TFs and the transcriptional output at defined loci [60,61], or by observing several molecular components (cofactors, Pol2, etc.) simultaneously [62,63]. Thus, there is hope that in the near future NEQ regulatory mechanisms could be inferred directly from measured molecular processes *in vivo*.

Lastly, we stress that certain successes of EQ models in eukaryotic systems cannot be used as evidence for equilibrium processes which would rule out NEQ models. As a pedagogic example, consider energy matrix (or, alternatively, position-weight-matrix (PWM)) models that are often used to describe the sequence-specific occupancy of TFs on DNA [11,64,65]. Although these models are typically rationalized or derived assuming thermodynamic equilibrium, their predictive success on real data does not necessarily imply that the real system is operating at equilibrium. First, these models are not models of regulation per se but of TF binding or occupancy only; a full regulation model additionally specifies how the information encoded by TF occupancies is integrated into transcriptional output, which could well be via a NEQ mechanism. Our high-specificity toy model (Figure 2a, left) is a case in point: the TF occupancy (of being either bound or active) is unaffected by the presence or absence of proof-reading (in both cases, it is given by k_+_/(k_+_ + k_−_), independently of the value of k_q_).

Second, even when energy matrix or PWM models provide great predictive power of expression from sequence assuming functions that are characteristic of equilibrium (e.g., expression is a logistic function of the predicted TF binding energy) [11,66], this does not exclude an equal or even better performance derived under alternative NEQ assumptions. In sum, we should be careful *not* to recognize predictive success of energy matrix models as evidence for EQ (and against NEQ) regulation; even in bacteria, where energy matrix models have been extremely successful, there is some evidence for NEQ processes that could dictate the unbinding of factors [8].

## Normative approach: navigating the space of non-equilibrium regulatory schemes

As the space of possible NEQ gene regulatory models is vast, can we turn to theory for guidance? Here we put forward a normative approach, which assess the functional relevance of regulatory schemes a priori [67]. One first identifies phenotypes of regulatory systems, which could have been evolutionarily selected for and which ideally can be measured experimentally. These phenotypes can be of various kinds: static regulatory phenotypes (expression amplitude, specificity, sensitivity), dynamic regulatory phenotypes (noise in gene expression, correlation or relaxation time of the gene expression output), or molecular phenotypes (TF residence time on the DNA, lifetimes of various other molecular complexes, etc.). The key idea is that some of these phenotypes can be experimentally estimated or bounded (e.g., TF residence time on the DNA), while others can be assumed to have been driven by evolution towards their optimal values (e.g., minimizing response time, minimizing noise, maximizing specificity etc.). Together, these assumptions define a constrained optimization problem whose solution identifies not a single model, but rather a class (or ensemble) of models—in terms of their structure and parameter domain—for which we have prior belief that they *may* be simultaneously biologically relevant and consistent with measured constraints and phenomenology [59]. Theory thereby suggests the ensemble to focus our attention and efforts on. Similar considerations could guide the optimal design of biochemical circuits [68,69].

Yet, surely, this approach must strongly depend on our assumptions about the regulatory phenotypes which are being optimized by evolution. We do not hide from this fact. Indeed, the functional relevance of regulatory phenotypes will depend on their biological context: what is beneficial for a developmental gene might be inappropriate for a housekeeping gene. For instance, the former might have been selected for high sensitivity (sharp spatial gene expression domain boundary during patterning) and short correlation time (fast response) [55,70], whereas the latter may have been selected for low sensitivity and long correlation time (promoting stability with respect to input changes). As with all modeling efforts, assumptions must be made. These assumptions (about evolutionary optimality), however, are no longer vague verbalizations at the end of a research paper, but become embodied in a mathematical formalism that, in the normative approach, we can parametrically vary and statistically assess, given the data. Ultimately, one could hope to identify a single high-level regulatory phenotype that subsumes the others, in order to propose a predictive theory for genetic regulation, paralleling the success of efficient coding in neuroscience [2].

**Figure 3:**
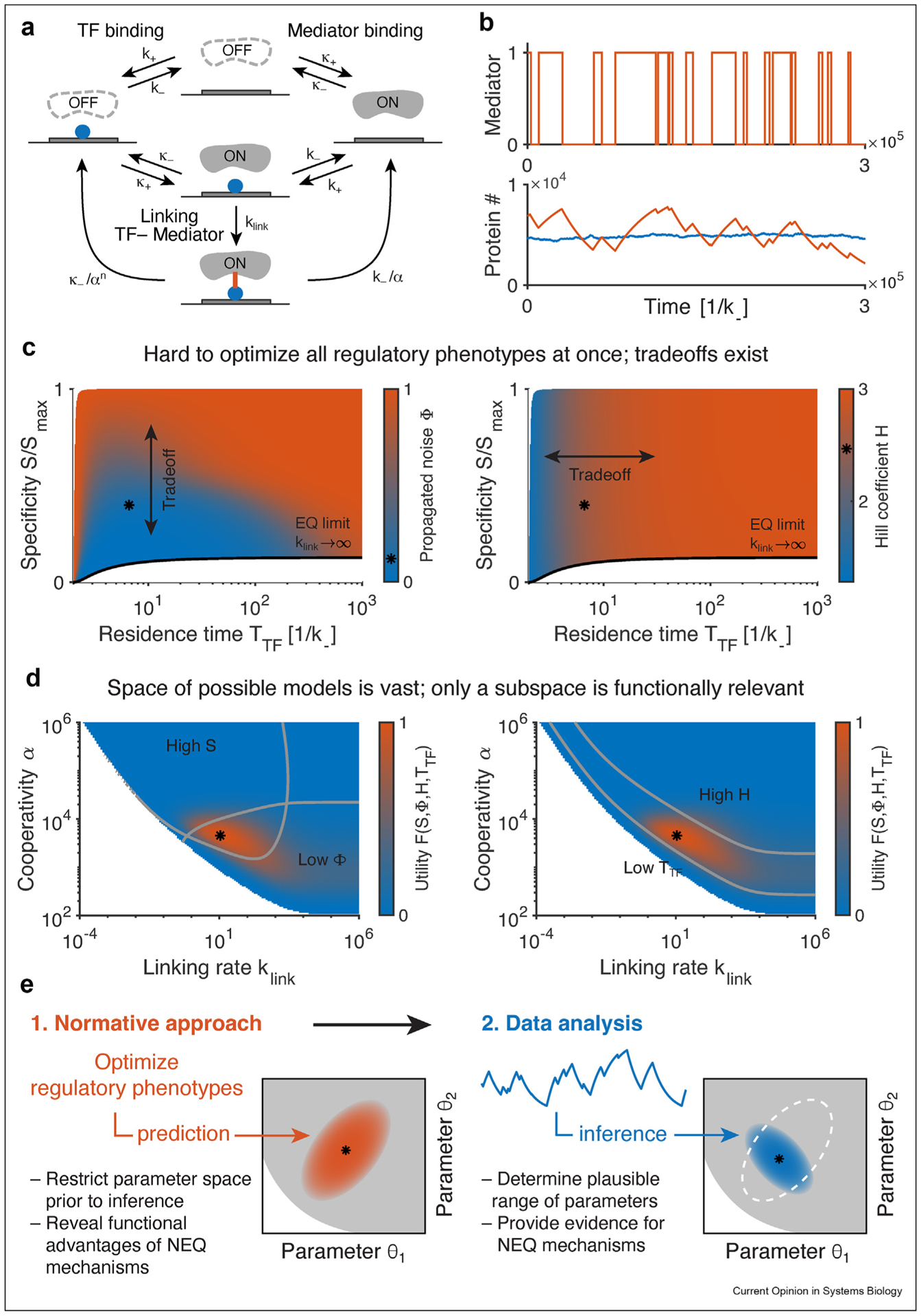
Normative approach helps us navigate a complex NEQ gene regulatory model. **(a)** Scheme of the Monod-Wyman-Changeux-like (MWC) model (here with N = 1 TF binding site, simplified from [44]) for a putative eukaryotic enhancer, describing TF un/binding (with rates k_−_ and k_+_) and Mediator un/binding (with rates κ_−_ and κ_+_), TF-Mediator interaction (parameter α), and a proof-reading step (with rate k_link_) by the formation of a link between the TF and the Mediator. For k_link_→∞, this model reduces to classic equilibrium MWC. **(b)** Stochastic realization of the model for N = 3 TF binding sites in EQ regime (blue, k_link_→∞) and NEQ regime (orange). (Top) Occupancy of the mediator-bound (expressing) state as a function of time. (Bottom) Protein counts simulated from the active state of the NEQ model show bursts due to slow activation dynamics, measured by the noise parameter Φ [44]. **(c)** Accessible regulatory phenotypes of the model at fixed average expression, such as specificity S, propagated noise Φ (left colorbar) and sensitivity H (right colorbar), as a function of TF residence time T_TF_. Black line corresponds to the EQ limit. Φ is in trade-off with S (high specificity implies high noise), while T_TF_ is in trade-off with H (high sensitivity implies high TF residence time). Thus, it is difficult to optimize all regulatory phenotypes at once. The star stands for one possibly relevant model that provides good improvement in S, high H, low Φ and low T_TF_. **(d)** Varying k_link_ and α enables sampling the regulatory space in (C). The colorbar reflects the magnitude of an arbitrary utility function (cf. [67]) of the combined phenotypes (increasing with S and H, decreasing with Φ and T_TF_). Gray curves represent equi-phenotype lines. Most parameter values lead to functionally unattractive models (blue region). Only a small subspace (orange region around the star) of models are functionally relevant as they simultaneously optimize multiple phenotypes. **(e)** By first optimizing the regulatory phenotypes, the normative approach can restrict the model or parameter space prior to inference, making model construction and sub-sequent inference of NEQ models more tractable.

Within the normative approach, one can thus numerically quantify how well a certain (inferred) model achieves a regulatory phenotype – or how far is it from the theoretical optimum. We review such an analysis [44] in Figure 3, which focuses on a possible model of eukaryotic gene regulation (Figure 3a), where TFs interact with the Mediator complex to drive expression (Figure 3b). The normative approach in this context allows us to compare various models quantitatively under identical conditions, for instance, at fixed average expression (cf. Figures 2d and 3c), or at (experimentally) fixed average TF residence time, and to compare NEQ models to their EQ limit (Figure 3c). One can discover functions that are inaccessible to EQ models, i.e., regulatory phenotypes that NEQ models can further minimize or maximize, as desired (cf. Figure 2 for specificity) [28,34]. One can furthermore discover tradeoffs (Figure 3c): increasing specificity might necessarily lead to higher gene expression noise [54,71]. Lastly, if a single regulatory phenotype is relevant, one can make testable *ab initio* predictions: optimization identifies model parameters which extremize the phenotype given measured constraints and these predictions can, at least in principle, be compared to direct inferences from data.

When the dust settles, the most important upshot of regulatory model space exploration might be the simple observation that the space of NEQ models is vast, and that most of that space is populated by dysfunctional models or models that do not outperform their equilibrium counterparts by any clear measure (Figure 2d right and 3d). At first glance, this prospect appears depressing. On the other hand, it means that if evolutionary adaptation *did* act to select for the chosen regulatory phenotypes, our normative approach will rule out most of the parameter space as deleterious, thereby focusing our models and attention into a small sub-space for the parameters and for the model selection (Figure 3e). From this perspective, such a normative approach should hold great promise for data modeling as well as its functional interpretation, especially when both can be performed within the same formal framework, as recently proposed [59].

## Future challenges

### Taming the complexity of NEQ models

Non-equilibrium mechanisms are free of all constraints on their rate parameters that stem from detailed balance. This leads to an unavoidable explosion of free parameters in NEQ models that complicates analysis and inference. One strategy for taming this complexity relies on ongoing progress in non-equilibrium statistical physics. Examples include: better understanding of the minimal energetic cost required to maintain a given NEQ steady state [72]; better understanding of the fluctuations in the different components of NEQ reaction networks and the constraints that these fluctuations must satisfy [73]; better understanding of the symmetries appearing in NEQ reaction network offering prospect of simplification [74]; and finally, development of relevant coarse-graining strategies [18].

Another strategy for taming the complexity is the normative approach that we have advocated here for [67]. As opposed to non-living matter, living systems have been evolutionarily selected for function, which must have resulted in implied selection for – and thus optimization of – various regulatory phenotypes. By identifying models that optimize various phenotypes we essentially restrict (or at least bias) the space of all possible models to a hopefully much smaller sub-space that is functionally relevant prior to further analysis or inference (Figure 3e). The only technical requirement is the ability to compute the regulatory phenotypes, but this can typically be done, at least numerically, from the master equation. We see the normative approach as complementary, not competing with, other ways to tame the complexity: time will tell whether these exciting theoretical advances actually shed light on real biochemical regulatory networks.

### Energetic costs of regulation

In eukaryotes, the energetic cost of putative NEQ regulation most likely represents only a small fraction of the total energy budget of a cell, and this cost might well be worth paying for. For instance, NEQ mechanisms (while requiring some energy expenditure) could alleviate even larger energetic costs of spurious or erroneous transcription and translation [24,44] that may be unavoidable for EQ regulatory schemes of lower specificity [65], sensitivity, or sub-optimal response timing [29,64].

Recent studies in yeast and mammalian cells have demonstrated that transcription and translation, when in excess, can represent a significant burden on global resources [75,76], which cells may try to avoid via optimal resource allocation. Optimal resource allocation arguments thus imply that even if the energy considerations ultimately govern the regulatory architecture, that happens indirectly: not by favoring regulatory schemes that are intrinsically energetically cheap (such as regulation at equilibrium), but rather, by favoring regulatory schemes that minimize the downstream energetic (or, perhaps more broadly, fitness) costs of mis-regulation. Further work is needed to quantify these costs and assess if they strengthen the case for NEQ regulatory schemes.

### Tantalizing cues for NEQ regulation

Certain reported features of eukaryotic regulation are hard to reconcile with equilibrium mechanisms. It is difficult to understand the high specificity of eukaryotic gene regulation through DNA binding where TFs recognize very short (6–10 bps) motifs, often with individually weak specificity [77,78]. High specificity could emerge from collective effects, either in equilibrium via various cooperative schemes [78,79] or out of equilibrium, e.g., via proof-reading [78,80–82]; it is important to note, however, that high equilibrium cooperativity does not automatically guarantee high specificity [65,71], a fact which often seems overlooked. In addition, the measured residence times of TFs on their specific binding sites tend to be short (a few seconds) and the binding events transient (e.g., as in “hit-and-run” regulation [83]), with the sequential ordering of TF and other cofactor (Mediator, P300, Brd4, etc.) arrivals playing a key role for proper Pol2 initiation and processive elongation [62,63,84]. Such a highly transient and sequential assembly of the pre-initiation complex, can hardly be accounted for by EQ models.

Lastly, the ever-changing chromatin landscape, the sequential accumulation of chromatin marks, and promoter state progression, lead to peaked inactive waiting-time distributions and suggest NEQ regulatory mechanisms [49,50,85]. More generally, gene regulatory steps beyond the TF binding to DNA could be out-of-equilibrium, complicating our search for experimental signatures of these interesting processes. An important task for the future is therefore to consider a totality of existing and new experimental results integratively, especially those results that have not even been collected with the explicit purpose to test the (non-) equilibrium regulation hypothesis.

## Simulation code for toy-models

The following Matlab functions are provided in a code repository accessible at https://github.com/BenjaminZoller/NEQ_Gene_Regulation_Review2022: (i) functions to compute the state rate matrix (Laplacian matrix of the master equation) for each model as a function of input parameters; (ii) generic functions to compute the various regulatory phenotypes and other features (waiting time distributions, fluxes, entropy production, etc.) from the state rate matrix; (iii) two functions to generate stochastic realizations of the models; (iv) four scripts to generate the different figures.

## Data Availability

Simulation code used for this research is freely available on GitHub, as referenced in the manuscript.

## Abbreviations

EQ: Equilibrium
NEQ: Non-equilibrium
TF: Transcription factor

## References

[1] Gnesotto FS, Mura F, Gladrow J, Broedersz CP: Broken detailed balance and non-equilibrium dynamics in living systems: a review. Rep Prog Phys 2018, 81, 66601. 10.1088/1361-6633/aab3ed 29504517

[2] Tkačik G, Bialek W: Information processing in living systems. Annu Rev Condens Matter Phys 2016, 7:89–117.

[3] Govern CC, Ten Wolde PR: Optimal resource allocation in cellular sensing systems. Proc Natl Acad Sci U S A 2014, 111: 17486–17491. 25422473 10.1073/pnas.1411524111 PMC4267345

[4.**] Anders A, Ghosh B, Glatter T, Sourjik V: Design of a MAPK signalling cascade balances energetic cost versus accuracy of information transmission. Nat Commun 2020, 11:3494. 32661402 10.1038/s41467-020-17276-4 PMC7359329

[5] Laughlin SB, De Ruyter Van Steveninck RR, Anderson JC: The metabolic cost of neural information. Nat Neurosci 1998, 1: 36–41. 10195106 10.1038/236

[6] Balasubramanian V, Sterling P: Receptive fields and functional architecture in the retina. J Physiol 2009, 587:2753–2767. 19525561 10.1113/jphysiol.2009.170704 PMC2718235

[7] Bintu L, Buchler NE, Garcia HG, Gerland U, Hwa T, Kondev J, Phillips R: Transcriptional regulation by the numbers: Models. Curr Opin Genet Dev 2005, 15:116–124. 15797194 10.1016/j.gde.2005.02.007 PMC3482385

[8] Hammar P, Walldén M, Fange D, Persson F, Baltekin Ö, Ullman G, Leroy P, Elf J: Direct measurement of transcription factor dissociation excludes a simple operator occupancy model for gene regulation. Nat Genet 2014, 46:405–408. 24562187 10.1038/ng.2905 PMC6193529

[9] Jones DL, Brewster RC, Phillips R: Promoter architecture dictates cell-to-cell variability in gene expression. Science 2014, 346:1533–1536. 80-. 25525251 10.1126/science.1255301 PMC4388425

[10.*] Morrison M, Razo-Mejia M, Phillips R: Reconciling kinetic and thermodynamic models of bacterial transcription. PLoS Comput Biol 2021, 17, e1008572. 33465069 10.1371/journal.pcbi.1008572 PMC7845990

[11] Barne SL, Belliveau NM, Ireland WT, Kinney JB, Phillips R: Mapping DNA sequence to transcription factor binding energy in vivo. PLoS Comput Biol 2019, 15, e1006226. 30716072 10.1371/journal.pcbi.1006226 PMC6375646

[12] Lagator M, Sarikas S, Steinrueck M, Toledo-Aparicio D, Bollback JP, Guet CC, Tkačik G: Predicting bacterial promoter function and evolution from random sequences. Elife 2022, 11:1–25. 10.7554/eLife.64543 PMC8791639 35080492

[13] Flamholz A, Phillips R, Milo R: The quantified cell. Mol Biol Cell 2014, 25:3497–3500. 25368429 10.1091/mbc.E14-09-1347 PMC4230611

[14] Lynch M, Marinov GK: The bioenergetic costs of a gene. Proc Natl Acad Sci U S A 2015, 112:15690–15695. 26575626 10.1073/pnas.1514974112 PMC4697398

[15] DeWane G, Salvi AM, DeMali KA: Fueling the cytoskeleton-links between cell metabolism and actin remodeling. J Cell Sci 2021, 134:jcs248385. 33558441 10.1242/jcs.248385 PMC7888749

[16] Wong F, Gunawardena J: Gene regulation in and out of equilibrium. Annu Rev Biophys 2020, 49:199–226. 32375018 10.1146/annurev-biophys-121219-081542

[17] Tkačik G, Mora T, Marre O, Amodei D, Palmer SE, Berry MJ, Bialek W: Thermodynamics and signatures of criticality in a network of neurons. Proc Natl Acad Sci USA 2015, 112: 11508–11513. 26330611 10.1073/pnas.1514188112 PMC4577210

[18] Yu Q, Zhang D, Tu Y: Inverse power law scaling of energy dissipation rate in nonequilibrium reaction networks. Phys Rev Lett 2021, 126, 80601. 10.1103/PhysRevLett.126.080601 PMC8286115 33709722

[19] Hill TL: Studies in irreversible thermodynamics IV. diagrammatic representation of steady state fluxes for unimolecular systems. J Theor Biol 1966, 10:442–459. 5964691 10.1016/0022-5193(66)90137-8

[20] Schnakenberg J: Network theory of microscopic and macroscopic behavior of master equation systems. Rev Mod Phys 1976, 48:571–585.

[21] Zia RKP, Schmittmann B: Probability currents as principal characteristics in the statistical mechanics of non-equilibrium steady states. J Stat Mech Theor Exp 2007, 2007: P07012. P07012.

[22] Ahsendorf T, Wong F, Eils R, Gunawardena J: A framework for modelling gene regulation which accommodates non-equilibrium mechanisms. BMC Biol 2014, 12:102. 25475875 10.1186/s12915-014-0102-4 PMC4288563

[23] Li C, Wang J: Landscape and flux reveal a new global view and physical quantification of mammalian cell cycle. Proc Natl Acad Sci U S A 2014, 111:14130–14135. 25228772 10.1073/pnas.1408628111 PMC4191801

[24] Molina N, Suter DM, Cannavo R, Zoller B, Gotic I, Naef F: Stimulus-induced modulation of transcriptional bursting in a single mammalian gene. Proc Natl Acad Sci U S A 2013, 110: 20563–20568. 24297917 10.1073/pnas.1312310110 PMC3870742

[25] Zoller B, Nicolas D, Molina N, Naef F: Structure of silent transcription intervals and noise characteristics of mammalian genes. Mol Syst Biol 2015, 11:823. 26215071 10.15252/msb.20156257 PMC4547851

[26] Scholes C, DePace AH, Sánchez Á: Combinatorial gene regulation through kinetic control of the transcription cycle. Cell Syst 2017, 4:97–108. e9. 28041762 10.1016/j.cels.2016.11.012 PMC5469051

[27] Hopefield JJ: Kinetic proofreading: a new mechanism for reducing errors in biosynthetic processes requiring high specificity. Proc Natl Acad Sci U S A 1974, 71:4135–4139. 4530290 10.1073/pnas.71.10.4135 PMC434344

[28] Cepeda-Humerez SA, Rieckh G, Tkačik G: Stochastic proof-reading mechanism alleviates crosstalk in transcriptional regulation. Phys Rev Lett 2015, 115, 248101. 26705657 10.1103/PhysRevLett.115.248101

[29] Galstyan V, Phillips R: Allostery and kinetic proofreading. J Phys Chem B 2019, 123:10990–11002. 31777251 10.1021/acs.jpcb.9b08380 PMC6995354

[30] Sartori P, Pigolotti S: Thermodynamics of error correction. Phys Rev X 2015, 5:1–9.

[31] Zhang J, Chen L, Zhou T: Analytical distribution and tunability of noise in a model of promoter progress. Biophys J 2012, 102: 1247–1257. 22455907 10.1016/j.bpj.2012.02.001 PMC3309289

[32] Rieckh G, Tkačik G: Noise and information transmission in promoters with multiple internal states. Biophys J 2014, 106: 1194–1204. 24606943 10.1016/j.bpj.2014.01.014 PMC4026790

[33] Tu Y: The nonequilibrium mechanism for ultrasensitivity in a biological switch: sensing by Maxwell’s demons. Proc Natl Acad Sci U S A 2008, 105:11737–11741. 18687900 10.1073/pnas.0804641105 PMC2575293

[34] Estrada J, Wong F, DePace A, Gunawardena J: Information integration and energy expenditure in gene regulation. Cell 2016, 166:234–244. 27368104 10.1016/j.cell.2016.06.012 PMC4930556

[35] Lang AH, Fisher CK, Mora T, Mehta P: Thermodynamics of statistical inference by cells. Phys Rev Lett 2014, 113, 148103. 25325665 10.1103/PhysRevLett.113.148103

[36] Sartori P, Tu Y: Free energy cost of reducing noise while maintaining a high sensitivity. Phys Rev Lett 2015, 115, 118102. 26406857 10.1103/PhysRevLett.115.118102 PMC4955832

[37] Li C, Cesbron F, Oehler M, Brunner M, Höfer T: Frequency modulation of transcriptional bursting enables sensitive and rapid gene regulation. Cell Syst 2018, 6:409–423.e11. 29454937 10.1016/j.cels.2018.01.012

[38] Cao Y, Wang H, Ouyang Q, Tu Y: The free-energy cost of accurate biochemical oscillations. Nat Phys 2015, 11: 772–778. 26566392 10.1038/nphys3412 PMC4638330

[39] Wierenga H, Ten Wolde PR, Becker NB: Quantifying fluctuations in reversible enzymatic cycles and clocks. Phys Rev E 2018, 97, 42404. 10.1103/PhysRevE.97.042404 29758603

[40] Roldán I, Parrondo JMR: Estimating dissipation from single stationary trajectories. Phys Rev Lett 2010, 105, 150607. 21230886 10.1103/PhysRevLett.105.150607

[41] Battle C, Broedersz CP, Fakhri N, Geyer VF, Howard J, Schmidt CF, MacKintosh FC: Broken detailed balance at mesoscopic scales in active biological systems. Science 2016, 352:604–607. 80-. 27126047 10.1126/science.aac8167 PMC8164727

[42.*] Martínez IA, Bisker G, Horowitz JM, Parrondo JMR: Inferring broken detailed balance in the absence of observable currents. Nat Commun 2019, 10:3542. 31387988 10.1038/s41467-019-11051-w PMC6684597

[43] Skinner DJ, Dunkel J: Estimating entropy production from waiting time distributions. arXiv 2021, 10.1103/PhysRevLett.127.198101 34797138

[44] Tu Y, Rappel WJ: Adaptation in living systems. Annu Rev Condens Matter Phys 2018, 9:183–205. 30057689 10.1146/annurev-conmatphys-033117-054046 PMC6060625

[45] Makarieva AM, Gorshkov VG, Li B-L, Chown SL, Reich PB, Gavrilov VM: Mean mass-specific metabolic rates are strikingly similar across life’s major domains: evidence for life’s metabolic optimum. Proc Natl Acad Sci USA 2008, 105: 16994–16999. 18952839 10.1073/pnas.0802148105 PMC2572558

[46] Rodenfels J, Neugebauer KM, Howard J: Heat oscillations driven by the embryonic cell cycle reveal the energetic costs of signaling. Dev Cell 2019, 48:646–658.e6. 30713074 10.1016/j.devcel.2018.12.024 PMC6414255

[47] Chen DTN, Heymann M, Fraden S, Nicastro D, Dogic Z: ATP consumption of eukaryotic flagella measured at a single-cell level. Biophys J 2015, 109:2562–2573. 26682814 10.1016/j.bpj.2015.11.003 PMC4699893

[48] Bel G, Munsky B, Nemenman I: The simplicity of completion time distributions for common complex biochemical processes. Phys Biol 2010, 7, 16003. 10.1088/1478-3975/7/1/016003 20026876

[49] Suter DM, Molina N, Gatfield D, Schneider K, Schibler U, Naef F: Mammalian genes are transcribed with widely different bursting kinetics. Science 2011, 332:472–474. 80-. 21415320 10.1126/science.1198817

[50] Harper CV, Finkenstädt B, Woodcock DJ, Friedrichsen S, Semprini S, Ashall L, Spiller DG, Mullins JJ, Rand DA, Davis JRE, : Dynamic analysis of stochastic transcription cycles. PLoS Biol 2011, 9, e1000607. 21532732 10.1371/journal.pbio.1000607 PMC3075210

[51] Cesbron F, Oehler M, Ha N, Sancar G, Brunner M: Transcriptional refractoriness is dependent on core promoter architecture. Nat Commun 2015, 6:6753. 25851692 10.1038/ncomms7753

[52.**] Henninger JE, Oksuz O, Shrinivas K, Sagi I, LeRoy G, Zheng MM, Andrews JO, Zamudio AV, Lazaris C, Hannett NM, : RNA-mediated feedback control of transcriptional condensates. Cell 2021, 184:207–225.e24. 33333019 10.1016/j.cell.2020.11.030 PMC8128340

[53] Zambrano S, Bianchi ME, Agresti A, Molina N: Interplay between stochasticity and negative feedback leads to pulsed dynamics and distinct gene activity patterns. Phys Rev E 2015, 92, 22711. 10.1103/PhysRevE.92.022711 26382436

[54.**] Grah R, Zoller B, Tkačik G: Nonequilibrium models of optimal enhancer function. Proc Natl Acad Sci U S A 2020, 117: 31614–31622. 33268497 10.1073/pnas.2006731117 PMC7749321

[55.*] Park J, Estrada J, Johnson G, Vincent BJ, Ricci-Tam C, Bragdon MD, Shulgina Y, Cha A, Wunderlich Z, Gunawardena J, : Dissecting the sharp response of a canonical developmental enhancer reveals multiple sources of cooperativity. Elife 2019, 8, e41266. 31223115 10.7554/eLife.41266 PMC6588347

[56] Eck E, Liu J, Kazemzadeh-Atoufi M, Ghoreishi S, Blythe S, Garcia HG: Quantitative dissection of transcription in development yields evidence for transcription factor-driven chromatin accessibility. Elife 2020, 9:1–99. 10.7554/eLife.56429 PMC7738189 33074101

[57] Zoller B, Little SC, Gregor T: Diverse spatial expression patterns emerge from unified kinetics of transcriptional bursting. Cell 2018, 175:835–847. e25. 30340044 10.1016/j.cell.2018.09.056 PMC6779125

[58] Munsky B, Li G, Fox ZR, Shepherd DP, Neuert G: Distribution shapes govern the discovery of predictive models for gene regulation. Proc Natl Acad Sci U S A 2018, 115:7533–7538. 29959206 10.1073/pnas.1804060115 PMC6055173

[59] Coulon A, Larson DR: Fluctuation analysis dissecting transcriptional kinetics with signal theory. Methods Enzymol 2016, 572:159–191. 27241754 10.1016/bs.mie.2016.03.017 PMC6300985

[60] Donovan BT, Huynh A, Ball DA, Patel HP, Poirier MG, Larson DR, Ferguson ML, Lenstra TL: Live-cell imaging reveals the interplay between transcription factors, nucleosomes, and bursting. EMBO J 2019, 38:e100809–e100818. 31101674 10.15252/embj.2018100809 PMC6576174

[61] Stavreva DA, Garcia DA, Fettweis G, Gudla PR, Zaki GF, Soni V, McGowan A, Williams G, Huynh A, Palangat M, : Transcriptional bursting and Co-bursting regulation by steroid hormone release pattern and transcription factor mobility. Mol Cell 2019, 75:1161–1177.e11. 31421980 10.1016/j.molcel.2019.06.042 PMC6754282

[62.**] Li J, Dong A, Saydaminova K, Chang H, Wang G, Ochiai H, Yamamoto T, Pertsinidis A: Single-molecule nanoscopy elucidates RNA polymerase II transcription at single genes in live cells. Cell 2019, 178:491–506.e28. 31155237 10.1016/j.cell.2019.05.029 PMC6675578

[63] Rosen GA, Baek I, Friedman LJ, Joo YJ, Buratowski S, Gelles J: Dynamics of RNA polymerase II and elongation factor Spt4/5 recruitment during activator-dependent transcription. Proc Natl Acad Sci U S A 2020, 117:32348–32357. 33293419 10.1073/pnas.2011224117 PMC7768755

[64] Stormo GD: Modeling the specificity of protein-DNA interactions. Quant Biol 2013, 1:115–130. 25045190 10.1007/s40484-013-0012-4 PMC4101922

[65] Ruan S, Stormo GD: Inherent limitations of probabilistic models for protein-DNA binding specificity. PLoS Comput Biol 2017, 13, e1005638. 28686588 10.1371/journal.pcbi.1005638 PMC5521849

[66] Segal E, Raveh-Sadka T, Schroeder M, Unnerstall U, Gaul U: Predicting expression patterns from regulatory sequence in Drosophila segmentation. Nature 2008, 451:535–540. 18172436 10.1038/nature06496

[67.**] Młynarski W, Hledík M, Sokolowski TR, Tkačik G: Statistical analysis and optimality of neural systems. Neuron 2021, 109: 1227–1241.e5. 33592180 10.1016/j.neuron.2021.01.020

[68] Fei C, Cao Y, Ouyang Q, Tu Y: Design principles for enhancing phase sensitivity and suppressing phase fluctuations simultaneously in biochemical oscillatory systems. Nat Commun 2018, 9:1–10. 29651016 10.1038/s41467-018-03826-4 PMC5897384

[69] Zhang D, Cao Y, Ouyang Q, Tu Y: The energy cost and optimal design for synchronization of coupled molecular oscillators. Nat Phys 2020, 16:95–100. 32670386 10.1038/s41567-019-0701-7 PMC7363412

[70] Tran H, Desponds J, Perez Romero CA, Coppey M, Fradin C, Dostatni N, Walczak AM: Precision in a rush: trade-offs between reproducibility and steepness of the hunchback expression pattern. PLoS Comput Biol 2018, 14, e1006513. 30307984 10.1371/journal.pcbi.1006513 PMC6198997

[71] Martins BMC, Swain PS: Trade-Offs and constraints in allosteric sensing. PLoS Comput Biol 2011, 7, e1002261. 22096453 10.1371/journal.pcbi.1002261 PMC3207937

[72] Horowitz JM, Zhou K, England JL: Minimum energetic cost to maintain a target nonequilibrium state. Phys Rev E 2017, 95, 42102. 10.1103/PhysRevE.95.042102 28505816

[73] Yan J, Hilfinger A, Vinnicombe G, Paulsson J: Kinetic uncertainty relations for the control of stochastic reaction networks. Phys Rev Lett 2019, 123, 108101. 31573304 10.1103/PhysRevLett.123.108101

[74] Biddle JW, Gunawardena J: Reversal symmetries for cyclic paths away from thermodynamic equilibrium. Phys Rev E 2020, 101, 62125. 10.1103/PhysRevE.101.062125 32688527

[75] Kafri M, Metzl-Raz E, Jona G, Barkai N: The cost of protein production. Cell Rep 2016, 14:22–31. 26725116 10.1016/j.celrep.2015.12.015 PMC4709330

[76.*] Frei T, Cella F, Tedeschi F, Gutiérrez J, Stan GB, Khammash M, Siciliano V: Characterization and mitigation of gene expression burden in mammalian cells. Nat Commun 2020, 11:4641. 32934213 10.1038/s41467-020-18392-x PMC7492461

[77] Wunderlich Z, Mirny LA: Different gene regulation strategies revealed by analysis of binding motifs. Trends Genet 2009, 25: 434–440. 19815308 10.1016/j.tig.2009.08.003 PMC3697852

[78] Friedlander T, Prizak R, Guet CC, Barton NH, Tkacik G: Intrinsic limits to gene regulation by global crosstalk. Nat Commun 2016, 7, 12307. 27489144 10.1038/ncomms12307 PMC4976215

[79] Mirny LA: Nucleosome-mediated cooperativity between transcription factors. Proc Natl Acad Sci U S A 2010, 107: 22534–22539. 21149679 10.1073/pnas.0913805107 PMC3012490

[80] Kribelbauer JF, Rastogi C, Bussemaker HJ, Mann RS: Low-affinity binding sites and the transcription factor specificity paradox in eukaryotes. Annu Rev Cell Dev Biol 2019, 35: 357–379. 31283382 10.1146/annurev-cellbio-100617-062719 PMC6787930

[81] Sánchez-Higueras C, Rastogi C, Voutev R, Bussemaker HJ, Mann RS, Hombría JCG: In vivo Hox binding specificity revealed by systematic changes to a single cis regulatory module. Nat Commun 2019, 10:3597. 31399572 10.1038/s41467-019-11416-1 PMC6689074

[82] Brodsky S, Jana T, Mittelman K, Chapal M, Kumar DK, Carmi M, Barkai N: Intrinsically disordered regions direct transcription factor in vivo binding specificity. Mol Cell 2020, 79:459–471. e4. 32553192 10.1016/j.molcel.2020.05.032

[83] Varala K, Li Y, Marshall-Colón A, Para A, Coruzzi GM: Hit-and-Run leaves its mark: catalyst transcription factors and chromatin modification. Bioessays 2015, 37:851–856. 26108710 10.1002/bies.201400205 PMC4548861

[84] Mir M, Reimer A, Haines JE, Li XY, Stadler M, Garcia H, Eisen MB, Darzacq X: Dense bicoid hubs accentuate binding along the morphogen gradient. Genes Dev 2017, 31: 1784–1794. 28982761 10.1101/gad.305078.117 PMC5666676

[85] Bintu L, Yong J, Antebi YE, McCue K, Kazuki Y, Uno N, Oshimura M, Elowitz MB: Dynamics of epigenetic regulation at the single-cell level. Science 2016, 351:720–724. 80-. 26912859 10.1126/science.aab2956 PMC5108652

